# Genomic Signatures of Adaptation to a Precipitation Gradient in Nigerian Sorghum

**DOI:** 10.1534/g3.118.200551

**Published:** 2018-08-14

**Authors:** Marcus O. Olatoye, Zhenbin Hu, Fanna Maina, Geoffrey P. Morris

**Affiliations:** Department of Agronomy, Kansas State University, Manhattan KS 66506

**Keywords:** Crop evolution, climate adaptation, flowering time, West Africa, genome-wide association studies

## Abstract

Evolution of plants under climatic gradients may lead to clinal adaptation. Understanding the genomic basis of clinal adaptation in crops species could facilitate breeding for climate resilience. We investigated signatures of clinal adaptation in the cereal crop sorghum (*Sorghum bicolor* L. [Moench]) to the precipitation gradient in West Africa using a panel (n = 607) of sorghum accessions from diverse agroclimatic zones of Nigeria. Significant correlations were observed between common-garden phenotypes of three putative climate-adaptive traits (flowering time, plant height, and panicle length) and climatic variables. The panel was characterized at >400,000 single nucleotide polymorphisms (SNPs) using genotyping-by-sequencing (GBS). Redundancy analysis indicated that a small proportion of SNP variation can be explained by climate (1%), space (1%), and climate collinear with space (3%). Discriminant analysis of principal components identified three genetic groups that are distributed differently along the precipitation gradient. Genome-wide association studies were conducted with phenotypes and three climatic variables (annual mean precipitation, precipitation in the driest quarter, and annual mean temperature). There was no overall enrichment of associations near *a priori* candidate genes implicated in flowering time, height, and inflorescence architecture in cereals, but several significant associations were found near *a priori* candidates including photoperiodic flowering regulators *SbCN12* and *Ma6*. Together, the findings suggest that a small (3%) but significant proportion of nucleotide variation in Nigerian sorghum landraces reflects clinal adaptation along the West African precipitation gradient.

Adaptation to environmental gradients can lead to evolution of clines, a continuous form of local adaptation (Slatkin 1973; Savolainen *et al.* 2013; Yoder *et al.* 2014). Climatic gradients that may shape trait adaptation include ultraviolet radiation, photoperiodicity, temperature, and precipitation (Hancock *et al.* 2011; Haussmann *et al.* 2012; Bastide *et al.* 2016). In the model plant Arabidopsis, clinal variation across latitude has been observed for seed dormancy, cold tolerance, height, and flowering time (Zhen and Ungerer 2008; Samis *et al.* 2012; Kronholm *et al.* 2012; Debieu *et al.* 2013). Likewise, in crop species, diffusion from tropical to temperate zones has led to clinal adaptation in flowering time (Ducrocq *et al.* 2008; Buckler *et al.* 2009; Wu *et al.* 2013; Kloosterman *et al.* 2013) and cold tolerance (Comadran *et al.* 2012; Ma *et al.* 2015). Local adaptation of traditional varieties has played a key role in smallholder crop production under adverse climatic conditions and low agricultural inputs (Vasconcelos *et al.* 2013). Locally-adapted crop landraces possess alleles that can be beneficial for the development of improved varieties to ensure food security under stressful climates (Zeven 1998; Soler *et al.* 2013; Lasky *et al.* 2015).

Understanding genetic diversity, population structure, and genotype-phenotype associations in crop landraces can guide germplasm conservation and breeding (Djè *et al.* 2000; Soler *et al.* 2013; Dwivedi *et al.* 2016). Recent advances in genotyping technology have facilitated studies of genomic diversity in crops, including studies of local and clinal adaptation using population and quantitative trait genomics (Myles *et al.* 2009; Morrell *et al.* 2012; Savolainen *et al.* 2013). Population genomics methods based on genome-wide patterns of nucleotide variation can identify loci with signatures of selection (Siol *et al.* 2010). These have been used to identify genomic targets of adaptation in many crops including maize (Gore *et al.* 2009; Hufford *et al.* 2012), rice (Meyer *et al.* 2016; Li *et al.* 2017), and sorghum (Morris *et al.* 2013; Mace *et al.* 2013). In sorghum, quantitative trait genomics approaches using mixed linear model have identified genomic regions associated with adaptive traits and climatic variables (Morris *et al.* 2013; Zhang *et al.* 2015; Lasky *et al.* 2015).

Sorghum (*Sorghum bicolor* L. [Moench]) is an essential staple cereal crop in dryland regions of the world (National Research Council 1996). It has adapted to a wide variety of climatic gradients and has abundant phenotypic variation for flowering time, plant morphology, and inflorescence morphology (Morris *et al.* 2013; Zhang *et al.* 2015; Lasky *et al.* 2015). Globally, the morphological types (botanical races) of sorghum are distributed according to precipitation zones, with open-panicle guinea types predominant in humid regions, semi-compact caudatum types predominant in semi-arid regions, and compact-panicle durra types predominant in arid regions (Doggett 1988; Morris *et al.* 2013). In West Africa, sorghum is found across a steep north-south precipitation gradient, ranging from semiarid grasslands bordering the Sahara Desert in the north (Sahelian zone), through subhumid savannah (Sudanian zone), to humid forest zones in the south (Guinean zone). These regions have been subject to major droughts for several millennia (Shanahan *et al.* 2009) and increased drought under climate change is expected to reduce sorghum yields in this region (Lobell *et al.* 2008).

The West African country of Nigeria is Africa’s most populous nation and its largest sorghum producer, with 5-10 million Mg of grain production per year (Nzeka and Akhidenor 2018). Sorghum is the major cereal in the northern Sudano-Sahelian region of Nigeria, which is characterized by prolonged dry seasons and short rainy seasons (National Research Council 1996). Sorghum, as a non-centric crop, has multiple centers of diversity and two of these overlap with the boundaries of Nigeria (Harlan 1971, 1992). The genetic diversity of Nigerian sorghum is poorly characterized compared to other African sorghum germplasm (Rao *et al.* 1985; Deu *et al.* 2008; Barro-Kondombo *et al.* 2010; Leiser *et al.* 2014). Identifying genomic regions underlying adaptation in Nigerian sorghum germplasm could facilitate the identification of adaptive traits and genetic diversity relevant to crop improvement.

Given that sorghum is distributed across the precipitation gradient in Nigeria, we hypothesized that Nigerian sorghum germplasm has been shaped by clinal adaptation. Under this hypothesis, we expect precipitation variables to be associated with both phenotype (putative climate-adaptive traits) and genotype (population structure and SNPs). Further, we expect that trait-associated and climate-associated genome regions will colocalize with genes involved in putative climate-adaptive traits. We investigated these predictions in a large panel of georeferenced Nigerian genebank accessions, which were previously phenotyped and which we genotyped at high-density using GBS. We characterized patterns of association among climatic, phenotypic, and genotypic variables and tested colocalization of associated genomic regions. Overall, the patterns are consistent with a small contribution of clinal adaptation shaping genomic variation in Nigerian sorghum.

## Materials and Methods

### Plant materials

Seeds for 553 Nigerian accessions were obtained from the USDA National Plant Germplasm System (NPGS) (https://www.ars-grin.gov/). Seedlings were raised in a greenhouse for two weeks and 50 mg of fresh leaf tissue was collected from each accession into 96-well plates. A control well was left empty on each plate. Leaf tissue was lyophilized (Labconco Freeze Dryer, Kansas City, MO, USA) for two days and then ground using 96-well plate plant tissue grinder (Retsch Mixer Mill, Haan, Germany). Genomic DNA was extracted using BioSprint 96 DNA Plant Kit (QIAGEN, Valencia CA, USA), quantified using Quant-iTTM PicoGreen dsDNA Assay Kit (ThermoFisher Scientific, Waltham MA, USA), then normalized to 10 ng/μl.

### Genotyping-by-sequencing

GBS was conducted on 553 Nigerian accessions using methods previously described (Elshire *et al.* 2011; Morris *et al.* 2013). Briefly, individual DNA samples were digested using *Ape*KI restriction enzyme (NEB R0643L) followed by ligation of barcode and common adapters ligation using T4 DNA ligase (NEB M0202L). Ligated libraries were pooled (96-plex libraries) then amplified by polymerase chain reaction (PCR). Purification of libraries was performed using QIAquick PCR purification kit (QIAGEN, Valencia CA, USA). Library size distribution was obtained using a Bioanalyzer (Agilent Technologies 2100, Santa Clara CA, USA). Four 96-plex libraries were pooled to generate 384-plex sequencing libraries. Libraries were sequenced using single end 100-cycle sequencing using Illumina HiSeq2500 (Illumina, San Diego CA, USA) at the University of Kansas Medical Center, Kansas City MO, USA.

**Figure 1:**
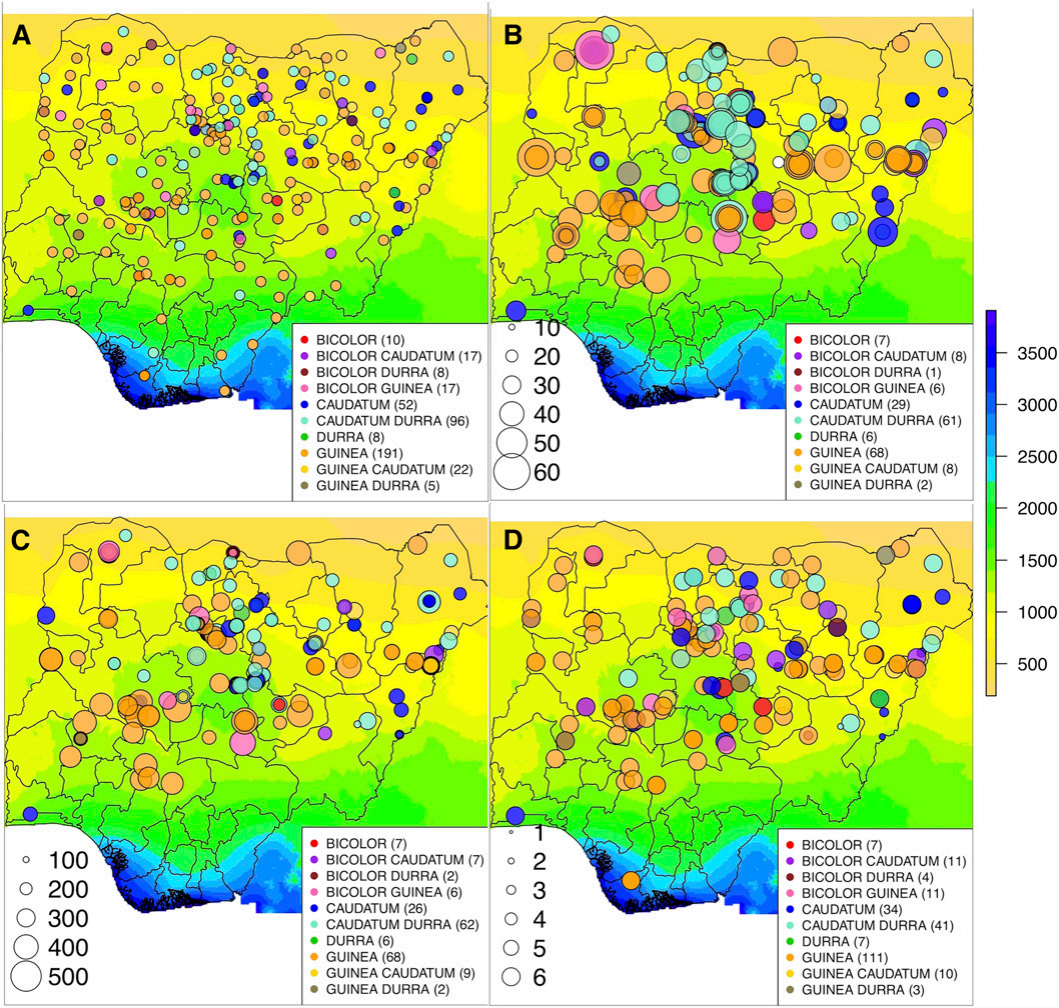
Geographic distribution of Nigerian accessions across precipitation zones. (A) Geographic distribution of georeferenced Nigerian accessions with known botanical race. (B) Panicle length variation across precipitation zones (size of circles commensurate to length of panicle in cm). (C) plant height variation across precipitation zones (size of circles commensurate to height of plant in cm). (D) flowering time variation across precipitation zones (size of circles commensurate to days until anthesis, 1 = earliest flowering and 6 = photoperiod sensitive). Background map is color-coded according to average annual precipitation (in mm).

Sequence reads for Nigerian germplasm were combined with published sequence reads obtained for 1943 accessions (Lasky *et al.* 2015). The published sequence data were composed of globally diverse sorghum landraces with major representation by accessions from Africa and Asia. They were obtained from the United States NPGS-GRIN and the International Crops Research Institute for the Semi-Arid Tropics (ICRISAT) gene banks. From these 1943 georeferenced global accessions, sequence information from 158 Nigerian accessions was obtained and combined with the Nigerian NPGS set. Duplicated accessions and accessions with sorghum conversion (SC) numbers (*i.e.*, with introgressions for early maturity and semi-dwarf genes) in the NPGS database were removed from the Nigerian germplasm. Thus, 607 Nigerian accessions (of which 443 were georeferenced; Figure 1A) and 1785 georeferenced global accessions were used for downstream analysis (Files S1 and S2). Reads were aligned to the sorghum reference genome v3.0 (McCormick *et al.* 2018) using Burrow Wheeler Alignment algorithm (Li and Durbin 2009). SNP calling was performed using TASSEL 5.0 GBS pipeline (Glaubitz *et al.* 2014). The SNPs were filtered for < 20% missingness, then missing data were imputed using BEAGLE 4.0 (Browning and Browning 2013).

### Climate and phenotype data

Climate data (average from 1960 to 1990) were obtained from WorldClim 1.4 using the Raster package in R (Hijmans *et al.* 2016) based on the coordinate (latitude and longitude) for each of the 443 georeferenced Nigerian accessions (File S1) and 1785 global accessions (File S2). As proxies for precipitation gradients that are hypothesized to affect Nigerian sorghum we investigated “annual precipitation” and “precipitation in the driest quarter”. Common garden passport data for flowering time, plant height, and panicle length for the Nigerian accessions were obtained from the USDA-NPGS Germplasm Resource Information Network database (https://www.ars-grin.gov/). The passport data were based on evaluations in one or more common garden experiments in tropical latitudes (Puerto Rico and St. Croix, 17-18°N), so best linear unbiased predictors (BLUPs) were estimated for each trait for each accession (File S1). A term for common garden was fit as random in the BLUP estimation model using *lmer* function in *LME4* package in R (Bates *et al.* 2014) as follows:

$$y_{i}=\mu +\gamma_{ij}+\varepsilon_{ijk}$$

where $y_{i}$ is the vector of phenotypic observation of the *i^th^* accession, $\gamma_{ij}$ is the *j^th^* common garden where *i^th^* accession was evaluated, and $\varepsilon_{ijk}$ is the residual or error term. Pearson correlations were calculated between BLUPs of three adaptive traits (flowering time, panicle length, and plant height), and environmental factors (latitude, temperature and precipitation). To reduce the influence of outlier sites with exceptional climatic variables, precipitation values of three geographical locations where sorghum is not commonly cultivated were removed. Analysis of variance (ANOVA) and Tukey HSD test in R were performed to identify precipitation differences among sites of origin for different genetic groups or botanical races.

### Redundancy analysis

Redundancy analysis (RDA) was performed separately for global and Nigerian germplasm sets using the *varpart* function in the R vegan package (Oksanen *et al.* 2017). A multivariate model was fit using the genomic data (431,698 SNPs for the global accession and 279,689 SNPs for the Nigerian accessions, filtered for monomorphic and singleton markers) as response variable. Ten WorldClim 1.4 climatic variables (annual mean temperature, mean temperature wettest quarter, mean temperature driest quarter, mean temperature warmest quarter, mean temperature coldest quarter, annual precipitation, precipitation wettest quarter, precipitation driest quarter, precipitation in the warmest quarter, and precipitation in the coldest quarter) and geographical variables (latitude and longitude, which we refer to as “space”) were fitted as predictor terms. The “space” term is included to account for isolation-by-distance (Lasky *et al.* 2012). To test the significance of the proportion of variation explained by climate collinear with space in the Nigerian germplasm, the proportion of variation explained was compared to the distribution from 1000 permuted data sets. In each stage of the permutation, individuals (genotypes) were randomized and RDA regression fitted and repeated 1000 times.

### Population structure and linkage disequilibrium analyses

Discriminant analysis of principal components (DAPC) was conducted with the *find clusters* function in Adegenet package in R (Jombart 2008; Jombart *et al.* 2010). Population differentiation (F_ST_) between DAPC groups was estimated using *–weir-fst-pop* parameter (Weir and Cockerham’s *F*_ST_) in VCFtools (Danecek *et al.* 2011). While nucleotide diversity within DAPC groups was estimated using *–window-pi* (1kb) in VCFtools. LD decay analysis for each DAPC group was performed by PopLDdecay (BGI-shenzhen 2017). For comparison with Nigerian germplasm, West African accessions were identified from the published global GBS data (Lasky *et al.* 2015). The published GBS data were composed of global accessions from 55 countries, predominantly representing landraces from sub-Saharan Africa and Asia. In the text, “global” refers to all accessions (including Nigerian and other West African accessions), unless otherwise noted that Nigerian or West African accessions have been removed.

Linkage disequilibrium decay for the genomic data for Nigerian, West African, and global germplasm was estimated by PopLDdecay (BGI-shenzhen 2017), with minor allele frequency parameter set at 0.05 and smoothing by the spline function in R. Principal component analysis (PCA) was performed using SNPrelate package in R (Zheng *et al.* 2012) with LD pruning threshold parameter set to 0.5 and minor allele frequency parameter set to 0.05. Neighbor-joining analysis was performed using TASSEL 5.0 and visualized in APE (Analyses of Phylogenetics and Evolution) package in R (Paradis *et al.* 2004). Population differentiation (F_ST_) between Nigerian, West African and global germplasm was evaluated using *–weir-fst-pop* parameter (Weir and Cockerham’s *F*_ST_) in VCFtools (Danecek *et al.* 2011). While nucleotide diversity within each germplasm, their inbreeding coefficients, and observed heterozygosity were estimated using *–window-pi* (1kb window), *–het*, and *–hardy* respectively, in VCFtools.

### Genome-wide association studies

Genome-wide association studies (GWAS) were performed using BLUPs of traits (panicle length, n = 330; plant height, n = 332; and flowering time, n = 412). After filtering the Nigerian data for a minimum minor allele frequency (MAF) of 0.03, a total of 149,342 SNPs were used in the GWAS analysis. First, a multi-locus mixed linear model (MLMM) (Segura *et al.* 2012) with a fixed population term (Q) and a random polygenic term (K) was used to perform GWAS for the phenotypic traits. PCA components (first three PCs) used for Q term were estimated using TASSEL 5.0 (Bradbury *et al.* 2007) and kinship matrix used for the polygenic term was derived from GAPIT (Lipka *et al.* 2012). Bonferroni correction of 2.6e-07 (α/number of markers, where α = 0.05) was used to determine the cut-off threshold for the phenotypic associations. A set of *a priori* candidate genes was compiled from Phytozome including known sorghum genes, and sorghum homologs of rice and maize genes known to be involved in inflorescence morphology, maturity, and plant height (n = 169; File S3).

### Genome scans

Three environmental variables (annual precipitation, precipitation in the driest quarter, and annual mean temperature) were used as proxies for the precipitation gradient (n = 443). A GLM, which does not include population structure and kinship terms, was used to perform an association scan for climatic variables to reduce false negatives (Bergelson and Roux 2010; Lasky *et al.* 2015). The top 1% outliers of the environmental associations were selected for enrichment analysis. Enrichment of *a priori* genes near association peak were performed using a chi-square test. Windows of 100 kb were used as conservative regions for colocalization between SNPs and *a priori* genes since LD decayed to background levels at > 100 kb. The genome wide Tajima’s *D* across 100 kb windows was tested using VCF tools in global germplasm (*D*_Global_), West African germplasm (*D*_WestAfrica_), and Nigerian germplasm (*D*_Nigeria_). Enrichment analysis for *a priori* genes was performed by testing whether the *D*_Nigeria_ 100 kb windows were significantly enriched for our *a priori* candidate genes relative to a set of random genes derived from the sorghum genome version 3 gff3 gene file from Phytozome (Goodstein *et al.* 2011; McCormick *et al.* 2018) for 1000 whole genome permutations.

### Data availability

Raw sequencing data are available from the NCBI Sequence Read Archive under project accession SRP132525 SNP genotype, phenotype, and geographic data are available at Dryad (doi:10.5061/dryad.g0141g7). All data are publicly available. File S1 contains detailed descriptions of Nigerian accessions, their passport data, georeference information, the BLUPs of phenotypes, climatic data, and DAPC groups. File S2 contains detailed descriptions of global accessions, their georeference information, and climatic data. File S3 contains *a priori* candidate genes list and literature sources. File S4 contains ANOVA and Tukey test results for race by precipitation analysis. File S5 contains detailed descriptions of *a priori* candidate genes associated with significant SNPs for MLMM and GLM GWAS results for the phenotypes. File S6 contains detailed descriptions of *a priori* candidate genes associated with outlier SNPs for GLM of environmental variables. File S7 contains detailed descriptions of *a priori* candidate genes associated with Nigerian germplasm Tajima’s *D* (*D*_Nigeria_) windows. Supplemental material available at Figshare: https://doi.org/10.25387/g3.6942986.

## Results

### Trait and environment correlations

The georeferenced sorghum accessions from Nigeria originated across a wide precipitation gradient (Figure 1A). Annual precipitation at the locations of origin ranges from < 500 mm/year for the northern Nigerian accessions (Sahelian zone) to > 3500 mm/year for the southern Nigerian accessions (Guinean zone). Analysis of variance indicates a significant difference in annual precipitation at sites of origin among the botanical races (*P*-value < 0.001). Significant differences were found between guinea and caudatum types (*P*-value < 0.05) and between guinea and durra-caudatum types (*P*-value < 0.01) (File S4) in precipitation differences among sites of origin.

**Figure 2:**
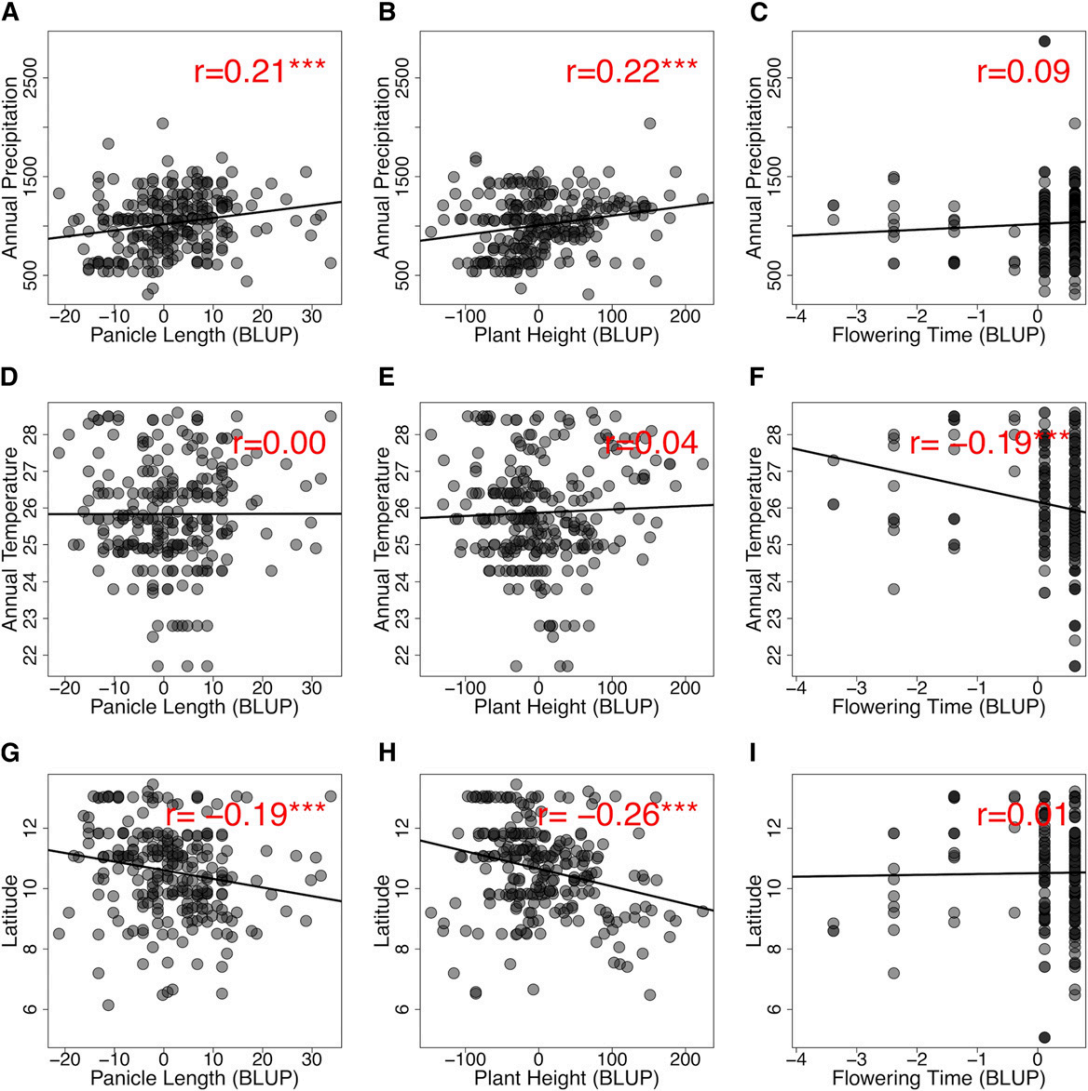
Relationships between traits and environmental variables in georeferenced germplasm. Pearson correlations between three environmental variables, annual mean precipitation (in mm) (A-C), annual temperature (in °C) (D-F), and latitude (G-I), *vs.* three phenotypic variables, panicle length (A, D, G), plant height (B, E, H), and flowering time (C, F, I) (n = 443). Phenotypes are BLUPs from common garden experiments. Significant correlation values are noted at 0.05, 0.01 and 0.001 (*, **, and ***).

The correlation of annual precipitation with common garden phenotypes of georeferenced Nigerian germplasm was investigated for three traits (panicle length, plant height, and flowering time) (Figure 1B-D). For comparison, we also considered correlations with annual mean temperature and latitude. Significant positive relationships with annual mean precipitation were observed for panicle length (Figure 2A; *r* = 0.21, *P*-value < 0.001) and plant height (Figure 2B; *r* = 0.22, *P*-value < 0.001) but not flowering time (Figure 2C). By contrast, annual mean temperature had no correlation with panicle length (Figure 2D) or plant height (Figure 2F) but a significant negative relationship with flowering time (Figure 2F; *r* = - 0.19; *P*-value < 0.001). Latitude had negative relationships with panicle length (Figure 2G; *r* = - 0.19, *P*-value < 0.001) and plant height (Figure 2H; *r* = - 0.26, *P*-value < 0.001), but no relationship with flowering time (Figure 2I). Among the traits, flowering time had significant positive relationships with both panicle length and plant height (Fig. S1; *r* = 0.32 and 0.41, respectively; *P*-values < 0.001). Among the environmental variables, annual mean precipitation had a significant negative relationship with latitude (Fig. S1; *r* = -0.86, *P*-value < 0.001) and a weaker but significant negative correlation with annual mean temperature (Fig. S1; *r* = -0.23, *P*-value < 0.001).

### Genome-wide nucleotide variation

To investigate genomic variation we developed a data set consisting of 431,698 SNPs genotyped across 2392 accessions (Nigeria, West Africa, and global). Most of the SNP variation in the three panels was rare; about 51% of the Nigerian genomic data were composed of SNPs with minor allele frequencies (MAF) < 0.01, 46% of the West African SNPs have MAF < 0.01, 36% of the global reference SNPs have MAF < 0.01, and 37% of global SNPs have MAF < 0.01 (Fig. S2A). The mean observed heterozygosity across loci in each of the germplasm is 0.02 (2%) (Fig. S2B). SNP density was higher in sub-telomeric regions and lower in sub-centromeric regions (Fig. S3). In the Nigerian germplasm (n = 607 accessions; Figure 1A), 279,689 SNPs were retained after removing monomorphic markers, singletons, and doubletons from the initial 431,698 SNPs. This corresponds to an average of 1 SNP per 2.7 kb. In the West African germplasm (n = 325 accessions), 311,786 SNPs were obtained after removing monomorphic markers, singletons, and doubletons.

The nucleotide diversity of Nigerian germplasm was similar to the West African and global germplasm. The average nucleotide diversity for global germplasm (π_global_) and West African germplasm (π_WestAfrica_) was 4.5 × 10^−4^. The nucleotide diversity in Nigerian germplasm (π_Nigeria_) was somewhat lower at 4.0 × 10^−4^. The average inbreeding coefficients were 0.83, 0.82, and 0.80 for global germplasm, West African germplasm, and Nigerian germplasm, respectively (all significantly different from each other at *P*-value < 0.01) (Fig. S2C). The *F*_ST_ between Nigerian and West African germplasm was 0.007 (Fig. S4A) and *F*_ST_ between Nigeria and global germplasm was 0.07 (Fig. S4B). The genome-wide average rate of linkage disequilibrium decay differed among the panels. Of the three germplasm sets, the global germplasm had the fastest LD decay rate by reducing to *r*^2^ = 0.1 at 20 kb (Fig. S5). LD decayed to half its initial value at 12 kb and *r*^2^ = 0.1 at 50 kb in the Nigerian germplasm. The West African germplasm had a slowest LD decay rate among the three sets, with *r*^2^ = 0.1 at 100 kb.

### Redundancy analysis

**Figure 3:**
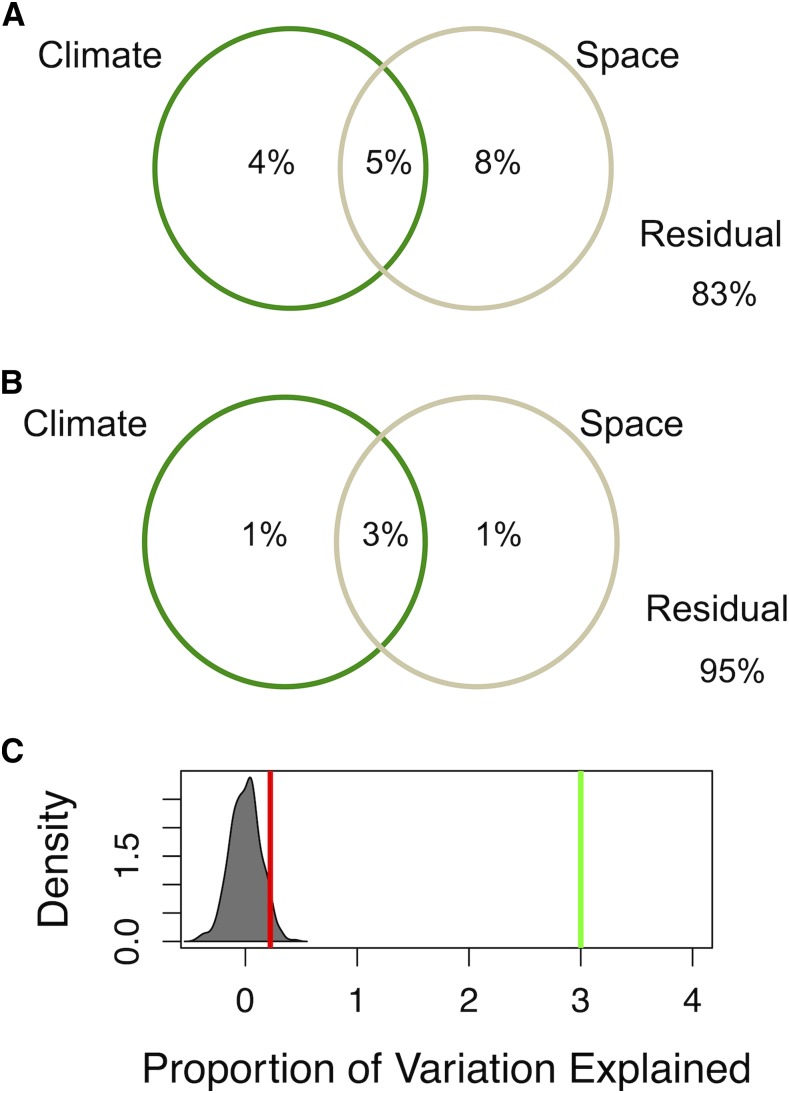
Redundancy analysis of SNP variation explained by climatic and spatial variables. Multivariate redundancy analysis showing the proportion of genotypic variation explained by climate variables (precipitation and temperature) and space (latitude and longitude) in (A) global germplasm and (B) Nigerian germplasm. (C) Density plot showing the distribution of proportion of variation explained by climate collinear with space obtained for 1000 permutations. The green line indicates the position of the proportion of variation explained by climate collinear with space and the red line indicates the 95^th^ percentile of the null distribution obtained by permutations.

To estimate the proportion of SNP variation that has been shaped by climate *vs.* geographic distance (space) we carried out a redundancy analysis, a form of multivariate regression. The proportion of SNP variation explained by climate and space in global germplasm was substantially greater than in Nigerian germplasm. In global germplasm, the proportion of variation explained by the 10 climate variables, space (latitude and longitude), and their combination together are 4%, 8%, and 5%, respectively (Figure 3A). By contrast, in Nigerian germplasm, climate and geographic variables explained a smaller proportion of the total SNP variation (Figure 3B); climate and space alone each explained 1% of the SNP variation, while climate collinear with space explained 3% of the SNP variation. The proportion of variation explained by climate collinear with space was significantly greater (*P* < 0.001) than the null distribution from geographically permuted data (Figure 3C).

### Population structure analysis of the Nigerian germplasm

To characterize the genetic structure of Nigerian germplasm in relation to global sorghum diversity, we conducted PCA and DAPC. In the PCA, the first PC explained about 6% of the variation while the second PC explained about 4% of the variation. The Nigerian accessions formed mostly separate clusters relative to the global accessions. The West African and Nigerian germplasm clustered together in most cases (Fig. S6A). Neighbor joining analysis also showed that Nigerian accessions and West African accessions cluster together, separately from the rest of the global germplasm (Fig. S7A). Clustering by botanical race was also observed in the Nigerian germplasm (Fig. S6B and Fig. S7B).

**Figure 4:**
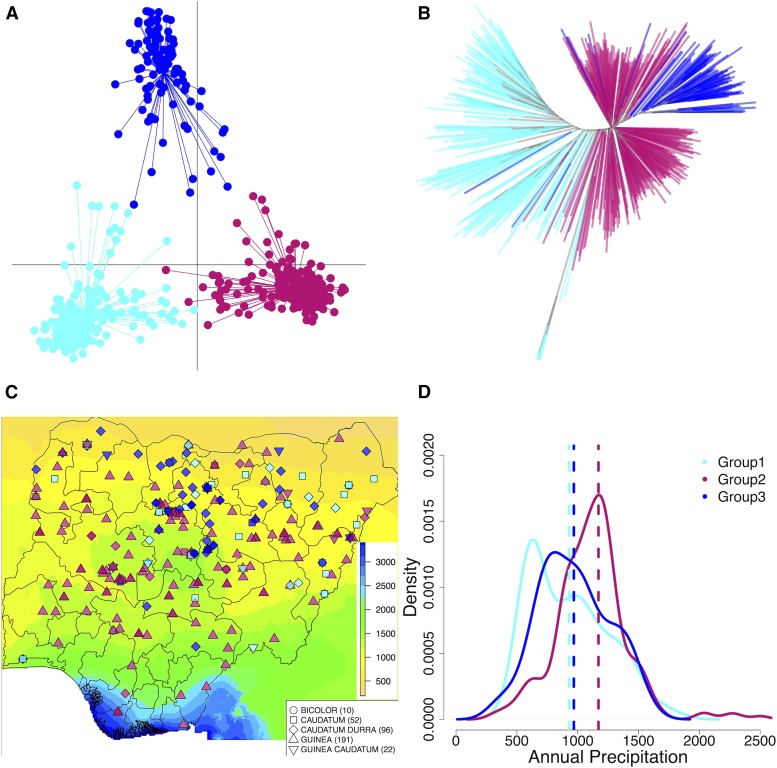
Genetic grouping of Nigerian germplasm in relation to the precipitation gradient. (A) Three genetic groups identified by the discriminant analysis of principal components (DAPC), (B) neighbor joining tree showing genetic relatedness among Nigerian accessions belonging to the three DAPC groups, (C) distribution of the georeferenced accessions based on their DAPC genetic groups across precipitation zones in Nigeria, and (D) density plots of precipitation distribution of georeferenced accessions based on their DAPC genetic groups. Group 2 accessions originate from locations with higher precipitation than Group 1 (*P*-value < 0.001) and Group 3 (*P*-value < 0.001).

DAPC analysis identified three genetic groups (Figure 4A–C). The DAPC groups were genetically differentiated from each other as follows: Group 1 *vs.* Group 2 (F_ST_ of 0.21), Group 1 *vs.* Group 3 (F_ST_ of 0.18), and Group 2 *vs.* Group 3 (F_ST_ of 0.22). Accessions in Group 2 originate from locations with higher precipitation than the accessions in Group 1 (*P*-value < 0.001) and Group 3 (*P*-value < 0.001) (Figure 4D). The average nucleotide diversity in 1kb windows for groups 1, 2, and 3 are 4.4 × 10^−4^, 3.3 × 10^−4^, and 3.8 × 10^−4^ respectively. Linkage disequilibrium (*r*^2^) level decayed to 0.1 at 30 kb in in Group 1, 80 kb in Group 2, and 90 kb in Group 3 (Fig. S8). Caudatum types are more predominant in Group 1, Guinea types are more predominant in Group 2, and Durra types are more predominant in Group 3 (Table 1).

**Table 1:** Distribution of sorghum botanical races among DAPC groups

Botanical Race	Group 1	Group 2	Group 3
Bicolor	6	3	1
Caudatum	39	7	6
Durra	4	1	3
Guinea	12	179	0
Bicolor Caudatum	9	5	3
Bicolor Durra	7	1	0
Bicolor Guinea	2	13	2
Caudatum Durra	28	11	57
Guinea Caudatum	5	5	4
Guinea Durra	1	1	1

In order to characterize variation among genetic groups identified by DAPC, ANOVA, and Tukey test were performed for putative adaptive traits and environmental variables. Significant difference in the distribution of precipitation and temperature gradient were found between DAPC Groups 1 and 3 (*P*-value < 0.01) and Groups 2 and 3 (*P*-value < 0.01). Also, there were significant differences between Groups 2 and 1 (*P*-value < 0.001) and Groups 2 and 3 (*P*-value < 0.001). We also found significant differences for putative adaptive traits between DAPC groups. For panicle length, all DAPC groups comparisons were different at *P*-value < 0.01. Significant differences were found for flowering time distribution between Groups 1 and 2 (*P*-value < 0.001). For plant height, significant differences were found between Groups 1 and 2 (*P*-value < 0.001) and Groups 2 and 3 (*P*-value < 0.001).

### Genome-wide association studies of phenotypes

**Figure 5:**
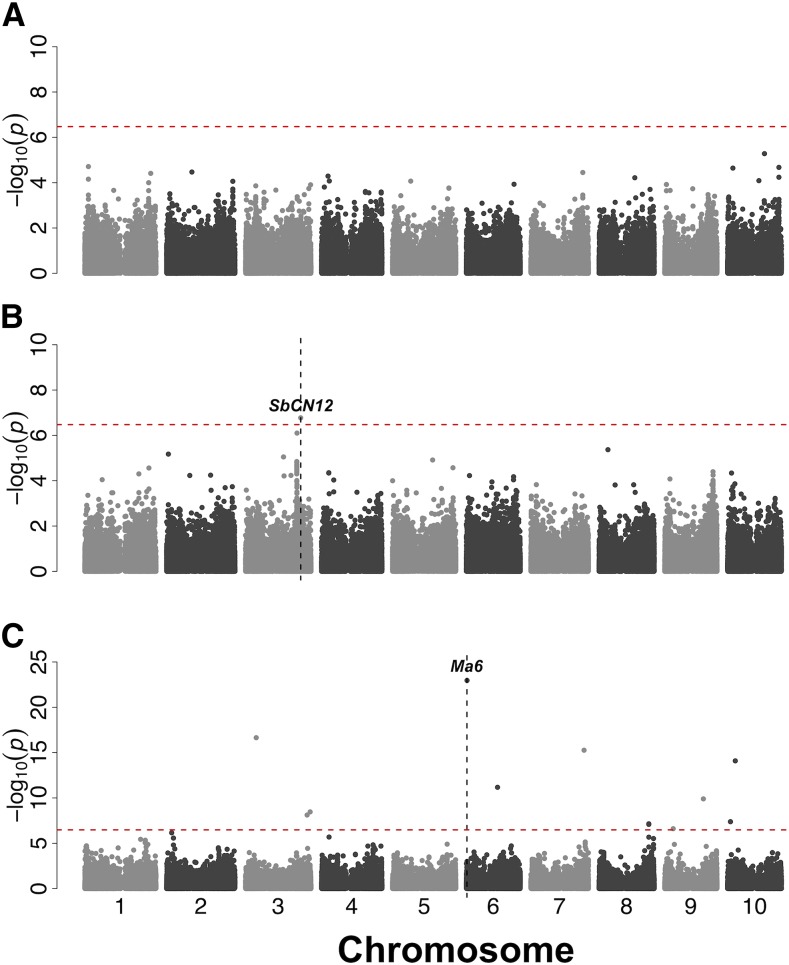
Genome wide association studies of phenotypes for putative adaptation traits. Genome-wide SNP associations for multi-locus mixed model with a fixed population term and a random polygenic term for (A) panicle length, (B) plant height, and (C) flowering time in Nigerian germplasm (n = 329, 331, and 411 respectively). Vertical dashed lines represent position of *a priori* candidate genes (*Ma6* and *SbCN12*) and found within 150 kb region of the significant association. Horizontal broken red lines signify the Bonferroni correction threshold (*P*-value < 0.05).

To identify genomic regions associated with phenotypic variation, we conducted MLMM GWAS for panicle length, plant height, and flowering time using BLUPs of common-garden phenotypes. Several genomic regions associated with the traits were identified and two *a priori* candidate genes fell within 100 kb (Figure 5, File S5). For panicle length no associations were significant at the Bonferroni threshold (Figure 5A). For plant height, a single significant association was observed on chromosome 3 (Figure 5B). The single significant association for plant height (S3_62675143, MAF = 0.29) colocalized with photoperiodic flowering gene *SbCN12* (Sobic.003G295300, 73 kb away) (Yang *et al.* 2014). For flowering time, nine significant associations were found on chromosomes 3, 6, 7, 9, and 10 (Figure 5C). The most significant flowering time association (S6_799609, MAF = 0.09) colocalized with the known sorghum flowering time and photoperiod sensitivity gene *Maturity6* (*Ma6/Ghd7*, Sobic.006G004400, 99 kb from gene) (Murphy *et al.* 2014). With a naive model (GLM GWAS), nominally significant associations were found on all chromosomes for panicle length, plant height, and flowering time, and a large number of these colocalized with *a priori* candidate genes (Fig. S9).

### Genome scans for adaptation

**Figure 6:**
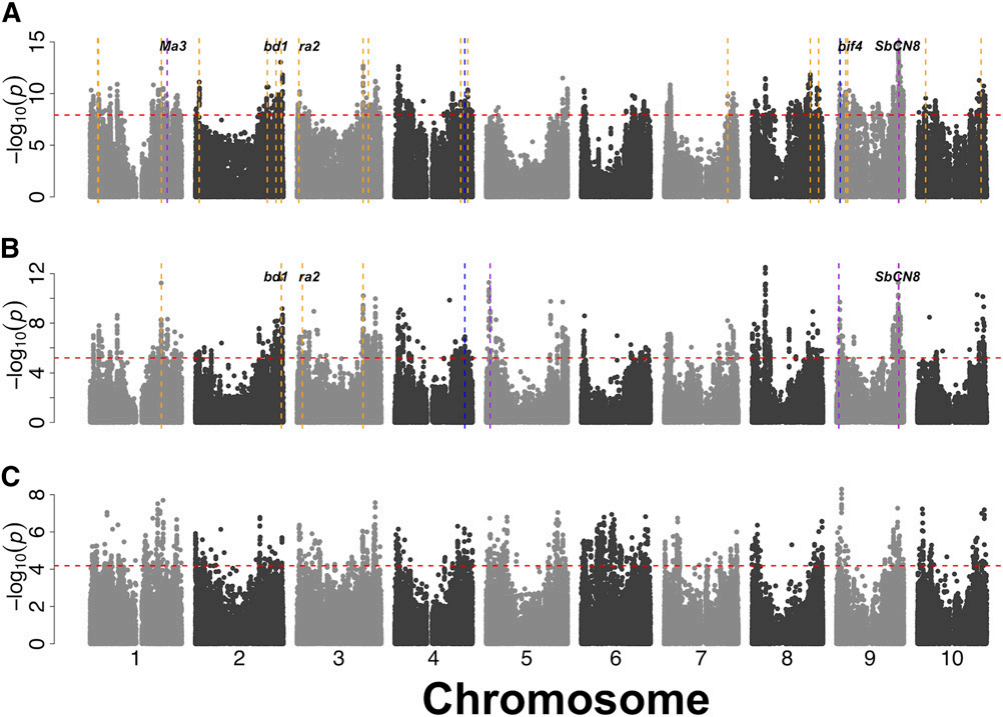
Genome wide association studies of climatic variables. Genome-wide SNP associations for general linear model of (A) annual mean precipitation, (B) precipitation in the driest quarter, (C) and annual mean temperature. Horizontal broken lines indicate the cutoff point of top 1% outliers. Vertical broken lines indicate the position of *a priori* candidate genes related to inflorescence morphology (orange), plant height (blue), and flowering time (purple), which are within 100 kb from associated SNPs.

Associations with environmental variables were used to investigate possible genomic signature of climate adaptation. GLM outliers (top 1% of associations) were identified on all chromosomes for “annual precipitation”, “precipitation in the driest quarter,” and “annual temperature” (Figure 6 A-D and File S6). Genome-wide, 15% and 6% of *a priori* candidate genes were localized within 100 kb of a 1% outlier SNP for “annual precipitation” and “precipitation in the driest quarter”, respectively (*vs.* 17% and 16% of all genes). Therefore, there is no enrichment of *a priori* candidate genes near environmental association outliers. A few of the *a priori* candidate genes that localize near 1% outlier SNPs are as follows. The SNP S9_54870238 (MAF = 0.36; 99^th^ percentile) associated with annual precipitation was 90 kb away from the regulator of photoperiodic flowering *SbCN8* (*Centroradialis8*, Sobic.009G199800). S9_8022437 (MAF = 0.49; 99^th^ percentile) associated with annual precipitation is 94 kb from the sorghum ortholog (Sobic.009G069700) of maize *barren inflorescence4* (*bif4*) (Galli *et al.* 2015). S3_4891237 and S3_4750963 (MAF = 0.31, 98^th^ percentile and MAF = 0.04, 99^th^ percentile) associated with annual precipitation and precipitation in the driest quarter were 100 kb from *Sbra2* (Sobic.003G052900), the sorghum ortholog of maize inflorescence gene *ramosa2* (*ra2*) (Brown *et al.* 2006).

Genome-wide pattern of Tajima’s *D* in the Nigerian germplasm (*D*_Nigeria_) across 1 kb windows ranged between -2.0 to 4.0 (Fig. S10 and Fig. S11). The average Tajima’s *D* value in the Nigerian germplasm (*D*_Nigeria_) was -0.2 while the average genome wide Tajima’s *D* (across 1 kb windows) in the global germplasm (*D*_Global_) and West African germplasm (*D*_WestAfrica_) were 0.1 and 0.2, respectively. Positive *D*_Nigeria_ windows were significantly enriched for *a priori* candidate genes compared to the expectation under a null distribution (Fig. S12 and File S7). Some of the *D*_Nigeria_ windows (Fig. S10) contain genes that control for flowering time and inflorescence development (File S7). For instance, the sorghum flowering time gene *Maturity6 (Ma6*, Sobic.006G004400) colocalized with the genome wide *D*_Nigeria_ scan window at 0.697 Mb (Tajima’s *D* = 1.48, 89^th^ percentile) on chromosome 6. *Maturity1* (*Ma1*, Sobic.006G057866) colocalized with the genome wide *D*_Nigeria_ scan window (Tajima’s *D* = 2.9; in the 98^th^ percentile) around 40 Mb on chromosome 6. The sorghum ortholog of *branched silkless1* (Sobic.002G411000), a maize spikelet meristem identity gene (Chuck *et al.* 2002), colocalized with the genome wide *D*_Nigeria_ scan window (Tajima’s *D* = 2.2 in the 94^th^ percentile) around 75.9 Mb on chromosome 2.

## Discussion

### Evidence for clinal adaptation to the precipitation gradient

Genome-wide studies of nucleotide variation can provide insights into patterns of genetic variation in crop landraces and the role of clinal adaptation in shaping this variation (Meyer *et al.* 2016). Overall, we found several lines of evidence that sorghum phenotypic variation across Nigeria has been shaped by clinal adaptation to precipitation. For panicle length, common garden variation in the Nigerian sorghum germplasm was correlated with annual precipitation (Figure 2, Fig. S1). Sorghum accessions originating from lower latitudes that have high precipitation had longer panicles than accessions originating from higher latitudes that have less precipitation (Figure 2A, 2G). The long panicle morphology is associated with open and lax primary branches, which is a key feature of guinea race which are predominant in humid to sub-humid regions of Nigeria (Figure 1A-B) and West Africa more generally (Deu *et al.* 2008; Barro-Kondombo *et al.* 2010; Lasky *et al.* 2015). This open panicle morphology is thought to allow airflow, reducing mold infection under high humidity (Doggett 1988), though this model has not been formally tested in diverse germplasm.

For plant height, sorghum accessions from lower latitudes associated with high precipitation were taller than sorghum accessions from higher latitudes associated with less precipitation (Figure 2B, 2H). This pattern is consistent with cross-species ecological studies of plant height, which identified precipitation as the best environmental predictor of within-species latitudinal variation of height (Moles *et al.* 2009). Given that higher latitudes in Nigeria have lower rainfall, reduced plant height and panicle length in dry regions may be an adaptation to increase yield stability under reduced water availability, as has been observed in West African pearl millet (Vigouroux *et al.* 2011). In our study, common garden variation in flowering time is associated with temperature but not precipitation at the location of origin. The negative relationship between flowering time and annual mean temperature (Figure 2F) suggests that sorghum in hot climates may flower early as an escape from high temperature and resulting water limitation (Tuinstra *et al.* 1997).

The significant proportion of nucleotide variation explained by climate collinear with space (3%; Figure 3) is consistent with clinal adaptation of sorghum in Nigeria. Redundancy analysis indicated that climate collinear with space explained more SNP variation in the Nigerian germplasm than either of climate and space (isolation-by-distance) alone. The finding that climate collinear with space explained more SNP variation than either climate or space alone is consistent with findings in global sorghum germplasm (Lasky *et al.* 2015) and regional germplasm in wild soybean (*Glycine soja*) (Leamy *et al.* 2016) and barley (*Hordeum vulgare*) landraces (Abebe *et al.* 2015). However, the proportion of SNP variation explained by climate collinear with space we observed in this study was much lower than what was observed in wild soybean and barley landraces (6–34% and 29–61%, respectively). Methodological differences that may contribute to the lower proportion of SNP variation explained in this study are the greater number of environmental variables used in the soybean and barley studies, and the use of ascertained SNPs in the soybean study.

Genome-wide nucleotide variation in Nigeria is also structured according to precipitation zones. The DAPC analysis identified genetic groups within the sorghum botanical races. These genetic groups showed differences in precipitation distributions (Figure 4C-D, Table 1). Group 1 was associated with the lowest annual mean precipitation, and composed predominantly of caudatum, caudatum intermediates and bicolor types and prevalent at higher latitudes in northeastern Nigeria characterized with lower annual precipitation. The northeastern part of Nigeria was classified as part of the center of diversity of caudatum and caudatum-durra (Harlan 1992). Group 2 was associated with higher annual precipitation distribution and more prevalent at lower latitudes. Most of the accessions in this group belong to the guinea and guinea intermediate racial types. Group 3 was predominantly made up of durra and caudatum-durra intermediates. Notably, there was a complete absence of the guinea race from this group. Consistent with the model that botanical races in sorghum are differentially adapted to precipitation-based agroclimatic zones (Harlan 1992; Morris *et al.* 2013), we found differences in botanical race distribution in precipitation zones (File S7). Precipitation distribution of guinea accessions was significantly different from precipitation distribution of caudatum (*P*-value < 0.05) and caudatum-durra races (*P*-value < 0.01).

Despite some evidence of clinal adaptation, we should note that the redundancy analysis (Figure 3) and theoretical considerations (Pavlidis *et al.* 2012; Meirmans 2015) suggest that bulk of the nucleotide variation observed in the Nigerian germplasm is neutral. In addition, the small proportion of phenotypic and genomic variation explained by the precipitation gradient (Figure 3B-C; Figure 2) suggest a modest role of clinal adaptation in shaping phenotypic diversity at the geographic scale of modern Nigeria. However, more detailed studies of panicle and vegetative morphology may reveal traits with stronger climate associations. Other factors that may have shaped the observed diversity patterns which we did not investigate include seed sharing based on ethnolinguistic grouping (Soler *et al.* 2013; Labeyrie *et al.* 2014) and historical processes of domestication and diffusion (Kimber *et al.* 2013; Morris *et al.* 2013). Consistent with a major role of cultural and historical factors shaping diversity at a Nigeria-wide scale, in the cases where multiple accessions were collected from single locations, multiple botanical races and genetic group were observed (Figure 1A, File S1).

### Identifying putative loci underlying adaptation

The combination of phenotypic association, environmental association, and selection scans can provide multiple lines of evidence for the involvement of particular loci in adaptation (Meyer *et al.* 2016). In the Nigerian sorghum germplasm, we observed some cases where *a priori* candidate genes related to adaptive traits colocalized with GWAS signals (Figure 5-6; File S5-S6). The colocalization of two sorghum photoperiod sensitivity genes (*SbCN12* and *Ma6*) with plant height and flowering time associations suggests that photoperiod sensitivity contributes to plant height and flowering time adaptation in Nigerian sorghum germplasm. This is consistent with previous studies that identified *SbCN12* and *Ma6/Ghd7* as major genes underlying natural variation in photoperiodic flowering in sorghum (Murphy *et al.* 2014; Yang *et al.* 2014; Bouchet *et al.* 2017). In sub-tropical latitudes, like the common gardens used by the USDA genebank (∼17° N), photoperiod sensitive sorghums are expected to have longer vegetative growth and attain greater heights than in tropical latitudes due to longer days at the higher latitudes (Murphy *et al.* 2014).

Given that strong association peaks for both environmental variables (annual precipitation and precipitation in the driest quarter; Figure 6) were found near *SbCN8*, this gene may be a candidate for clinal adaptation to the precipitation gradient in West Africa. The colocalization of inflorescence genes (*ra2* and *bif4*) with associations for annual precipitation (Figure 6A; File S5-S6) is consistent with an adaptive role of inflorescence morphology across the precipitation gradient (Harlan 1992; Morris *et al.* 2013). Accounting for population structure in GWAS models when mapping phenotypes that are correlated with population structure can lead to false negatives (Bergelson and Roux 2010; Lasky *et al.* 2015). Notably there were no significant associations for panicle length after accounting for population structure (MLM; Figure 5A). This finding is consistent with the expectation that climate-associated traits will be confounded with population structure and there will be little power to map the genetic basis of these traits using mixed model association (Brachi *et al.* 2011). Genetic dissection of panicle morphology and other putative clinal adaptation traits should be more effective with a multi-parent mapping strategy that breaks up confounding population structure, such as nested association mapping (Buckler *et al.* 2009; Bouchet *et al.* 2017).

Long-term stable clinal adaptation is expected to be reflected in genomic signals of balancing selection (Novembre and Rienzo 2009; Yoder *et al.* 2014). Consistent with the phenotypic evidence of clinal trait adaptation (Figure 2A, 2B, 2F), there was evidence of balancing selection from genome-wide enrichment of *D*_Nigeria_ windows at *a priori* candidate genes for flowering time, plant height, and inflorescence morphology (Fig. S11 and Fig. S12). Further functional studies of candidate genes will be needed to establish if these candidate genes have a role in climate adaptation (Kesari *et al.* 2012; Romero Navarro *et al.* 2017). Resequencing of georeferenced germplasm should facilitate the identification of putative functional variants underlying clinal adaptation (Gore *et al.* 2009; Meyer *et al.* 2016). Demographic effects due to historical processes of domestication and differentiation affect the pattern of diversity under neutrality (Molina *et al.* 2011; Meyer and Purugganan 2013). While it is suspected that sorghum has a complex domestication history based on morphology (Harlan 1992), population structure (Deu *et al.* 2006), and shattering gene haplotypes (*Shattering1*) (Olsen 2012), the demographic scenarios have not been formally described or evaluated. Therefore, further study on demographic history in sorghum will be valuable to identify which genomic outlier loci are most likely to represent signatures of selection.

If variation at genes involved in putative adaptive traits (flowering time, plant height, and panicle length) underlie clinal variation, then we expect significant enrichment of the *a priori* genes with environment associations (Rellstab *et al.* 2015). However, there was no enrichment *of a priori* candidate genes near associations with “precipitation in the driest quarter” or “annual mean precipitation”. An alternative to the single-trait GWAS and colocalization approach used in this study would be a multi-trait GWAS approach (Korte *et al.* 2012; Zhou and Stephens 2014) simultaneously considering traits and environmental variables. However, biological interpretation of the synthetic or “eigen” traits may remain a challenge until more corroborating functional genetic data are available (Banerjee *et al.* 2008).

### Resources for genomic-enabled breeding of clinal adaptation

The Nigerian germplasm harbors abundant nucleotide polymorphism (90% of the global nucleotide polymorphism based on the π_Nigeria_ *vs.* π_global_), consistent with West Africa as a center of diversity for sorghum (Harlan 1992). The high diversity could be a result of ancestral diversity in the Nigerian sorghum, and/or gene flow and diffusion from other regions of Africa (Westengen *et al.* 2014). Population structure and neighbor-joining tree analysis showed that majority of the Nigerian accessions and West African accessions clustered together (Fig. S6 and Fig. S7). The Nigerian germplasm was 10 times less differentiated from West African germplasm compared to the rest of global sorghum germplasm (*F*_ST_ Nigeria-West Africa = 0.007 and *F*_ST_ Nigeria-Global = 0.07). Low level of differentiation among regional germplasm in sorghum has been attributed to gene flow from human migration and agricultural trade (Menkir *et al.* 1997).

Characterization of LD patterns is critical for interpretation of genome scans since the local extent of LD decay determines resolution of mapping and long-range LD creates spurious associations (Myles *et al.* 2009). LD decay rate in Nigerian germplasm (half of maximum value at 12 kb) was slower than LD decay estimated in the global sorghum association panel (half of maximum value at 1 kb) (Morris *et al.* 2013). The global sorghum association panel may capture more historical recombination than the Nigerian germplasm because of its greater geographic diversity. The reduced long-range LD decay in the Nigerian germplasm compared to the West African germplasm (Fig. S5) may be due to the smaller geographic scale, which should reduce long range LD due to isolation-by-distance (Brachi *et al.* 2011). Given the observed LD decay rates, the mapping resolution for genome-wide scans in the Nigerian germplasm is expected to be less than in global sorghum panels but greater than in West African regional panels. Overall, the modest LD decay rate and high genetic diversity in the Nigerian germplasm make it suitable for genome-wide association studies.

The application of genomics for crop improvement and plant genetic resource management is still in the early stage for most national agricultural research systems in sub-Saharan Africa, including Nigeria and neighboring countries (Ezeaku and Gupta 2004; Leiser *et al.* 2014; Yohannes *et al.* 2015). The genomic resources developed in this study represent a step toward genomics-enabled breeding and germplasm management for sorghum landraces in Nigeria. The resources developed include a genome-wide catalog of SNP variation, a description of geographic population structure, and estimates of genetic properties including nucleotide diversity and LD decay. The genomic signatures of clinal adaptation identified in this study, if validated in managed stress and multi-environment mapping studies (Cooper *et al.* 2014; Lasky *et al.* 2015), could be another resource to facilitate genomics-enabled breeding for climate-resilience in West Africa.

## References

[bib1] Abebe T. D. Naz A. A. Léon J., 2015 Landscape genomics reveal signatures of local adaptation in barley (Hordeum vulgare L.). Front. Plant Sci. 6: 813 10.3389/fpls.2015.00813 26483825 PMC4591487

[bib2] Banerjee S. Yandell B. S. Yi N., 2008 Bayesian Quantitative Trait Loci Mapping for Multiple Traits. Genetics 179: 2275–2289. 10.1534/genetics.108.088427 18689903 PMC2516097

[bib3] Barro-Kondombo C. Sagnard F. Chantereau J. Deu M. vom Brocke K., 2010 Genetic structure among sorghum landraces as revealed by morphological variation and microsatellite markers in three agroclimatic regions of Burkina Faso. Theor. Appl. Genet. 120: 1511–1523. 10.1007/s00122-010-1272-2 20180097

[bib4] Bastide H. Lange J. D. Lack J. B. Yassin A. Pool J. E., 2016 A variable genetic architecture of melanic evolution in Drosophila melanogaster. Genetics genetics.116.192492 10.1534/genetics.116.192492

[bib5] Bates, D., M. Maechler, B. Bolker, S. Walker, R. H. B. Christensen *et al.*, 2014 lme4: Linear mixed-effects models using Eigen and S4.

[bib6] Bergelson J. Roux F., 2010 Towards identifying genes underlying ecologically relevant traits in Arabidopsis thaliana. Nat. Rev. Genet. 11: 867–879. 10.1038/nrg2896 21085205

[bib7] Bouchet S. Olatoye M. O. Marla S. R. Perumal R. Tesso T., 2017 Increased power to dissect adaptive traits in global sorghum diversity using a nested association mapping population. Genetics 206: 573–585. 10.1534/genetics.116.198499 28592497 PMC5499173

[bib8] Brachi B. Morris G. P. Borevitz J. O., 2011 Genome-wide association studies in plants: the missing heritability is in the field. Genome Biol. 12: 232 10.1186/gb-2011-12-10-232 22035733 PMC3333769

[bib9] Bradbury P. J. Zhang Z. Kroon D. E. Casstevens T. M. Ramdoss Y., 2007 TASSEL: software for association mapping of complex traits in diverse samples. Bioinformatics 23: 2633–2635. 10.1093/bioinformatics/btm308 17586829

[bib10] Brown P. Klein P. Bortiri E. Acharya C. Rooney W., 2006 Inheritance of inflorescence architecture in sorghum. Theor. Appl. Genet. 113: 931–942. 10.1007/s00122-006-0352-9 16847662

[bib12] Browning B. L. Browning S. R., 2013 Improving the accuracy and efficiency of identity-by-descent detection in population data. Genetics 194: 459–471. 10.1534/genetics.113.150029 23535385 PMC3664855

[bib13] Buckler E. S. Holland J. B. Bradbury P. J. Acharya C. B. Brown P. J., 2009 The genetic architecture of maize flowering time. Science 325: 714–718. 10.1126/science.1174276 19661422

[bib14] Chuck G. Muszynski M. Kellogg E. Hake S. Schmidt R. J., 2002 The control of spikelet meristem identity by the branched silkless1 gene in maize. Science 298: 1238–1241. 10.1126/science.1076920 12424380

[bib15] Comadran J. Kilian B. Russell J. Ramsay L. Stein N., 2012 Natural variation in a homolog of Antirrhinum CENTRORADIALIS contributed to spring growth habit and environmental adaptation in cultivated barley. Nat. Genet. 44: 1388–1392. 10.1038/ng.2447 23160098

[bib16] Cooper M. Messina C. D. Podlich D. Totir L. R. Baumgarten A., 2014 Predicting the future of plant breeding: complementing empirical evaluation with genetic prediction. Crop Pasture Sci. 65: 311–336. 10.1071/CP14007

[bib17] Danecek P. Auton A. Abecasis G. Albers C. A. Banks E., 2011 The variant call format and VCFtools. Bioinformatics 27: 2156–2158. 10.1093/bioinformatics/btr330 21653522 PMC3137218

[bib18] Debieu M. Tang C. Stich B. Sikosek T. Effgen S., 2013 Co-variation between seed dormancy, growth rate and flowering time changes with latitude in Arabidopsis thaliana. PLoS One 8: e61075 10.1371/journal.pone.0061075 23717385 PMC3662791

[bib19] Deu M. Rattunde F. Chantereau J., 2006 A global view of genetic diversity in cultivated sorghums using a core collection. Genome 49: 168–180. 10.1139/g05-092 16498467

[bib20] Deu M. Sagnard F. Chantereau J. Calatayud C. Hérault D., 2008 Niger-wide assessment of in situ sorghum genetic diversity with microsatellite markers. Theor. Appl. Genet. 116: 903–913. 10.1007/s00122-008-0721-7 18273600

[bib21] Djè Y. Heuertz M. Lefèbvre C. Vekemans X., 2000 Assessment of genetic diversity within and among germplasm accessions in cultivated sorghum using microsatellite markers. Theor. Appl. Genet. 100: 918–925. 10.1007/s001220051371

[bib22] Doggett H., 1988 Sorghum, 2d. ed. Tropical agricultural series. Longman Scientific, Essex, UK.

[bib23] Ducrocq S. Madur D. Veyrieras J.-B. Camus-Kulandaivelu L. Kloiber-Maitz M., 2008 Key impact of Vgt1 on flowering time adaptation in maize: Evidence from association mapping and ecogeographical information. Genetics 178: 2433–2437. 10.1534/genetics.107.084830 18430961 PMC2323828

[bib24] Dwivedi S. L. Ceccarelli S. Blair M. W. Upadhyaya H. D. Are A. K., 2016 Landrace germplasm for improving yield and abiotic stress adaptation. Trends Plant Sci. 21: 31–42. 10.1016/j.tplants.2015.10.012 26559599

[bib25] Elshire R. J. Glaubitz J. C. Sun Q. Poland J. A. Kawamoto K., 2011 A robust, simple genotyping-by-sequencing (GBS) approach for high diversity species. PLoS One 6: e19379 10.1371/journal.pone.0019379 21573248 PMC3087801

[bib26] Ezeaku I. E. Gupta S. C., 2004 Development of sorghum populations for resistance to Striga hermonthica in the Nigerian Sudan Savanna. Afr. J. Biotechnol. 3: 324–329. 10.5897/AJB2004.000-2059

[bib27] Galli M. Liu Q. Moss B. L. Malcomber S. Li W., 2015 Auxin signaling modules regulate maize inflorescence architecture. Proc. Natl. Acad. Sci. USA 112: 13372–13377. 10.1073/pnas.1516473112 26464512 PMC4629326

[bib28] Glaubitz J. C. Casstevens T. M. Lu F. Harriman J. Elshire R. J., 2014 TASSEL-GBS: A high capacity genotyping by sequencing analysis pipeline. PLoS One 9: e90346 10.1371/journal.pone.0090346 24587335 PMC3938676

[bib29] Goodstein D. M. Shu S. Howson R. Neupane R. Hayes R. D., 2011 Phytozome: a comparative platform for green plant genomics. Nucleic Acids Res. 40: D1178–D1186. 10.1093/nar/gkr944 22110026 PMC3245001

[bib30] Gore M. A. Chia J.-M. Elshire R. J. Sun Q. Ersoz E. S., 2009 A first-generation haplotype map of maize. Science 326: 1115–1117. 10.1126/science.1177837 19965431

[bib31] Hancock A. M. Witonsky D. B. Alkorta-Aranburu G. Beall C. M. Gebremedhin A., 2011 Adaptations to climate-mediated selective pressures in humans. PLoS Genet. 7: e1001375 10.1371/journal.pgen.1001375 21533023 PMC3080864

[bib32] Harlan J. R., 1971 Agricultural origins: centers and noncenters. Science 174: 468–474. 10.1126/science.174.4008.468 17745730

[bib33] Harlan J. R., 1992 Crops and Man, American Society of Agronomy, Madison, Wisconsin.

[bib34] Haussmann B. I. G. Fred Rattunde H. Weltzien-Rattunde E. Traoré P. S. C. vom Brocke K., 2012 Breeding strategies for adaptation of pearl millet and sorghum to climate variability and change in West Africa. J. Agron. Crop Sci. 198: 327–339. 10.1111/j.1439-037X.2012.00526.x

[bib35] Hijmans, R. J., J. van Etten, J. Cheng, M. Mattiuzzi, M. Sumner *et al.*, 2016 *raster: Geographic Data Analysis and Modeling*.

[bib36] Hufford M. B. Xu X. van Heerwaarden J. Pyhäjärvi T. Chia J.-M., 2012 Comparative population genomics of maize domestication and improvement. Nat. Genet. 44: 808–811. 10.1038/ng.2309 22660546 PMC5531767

[bib37] Jombart T., 2008 Adegenet: A R Package for the Multivariate Analysis of Genetic Markers. Bioinformatics 24: 1403–1405. 10.1093/bioinformatics/btn129 18397895

[bib38] Jombart T. Devillard S. Balloux F., 2010 Discriminant analysis of principal components: a new method for the analysis of genetically structured populations. BMC Genet. 11: 94 10.1186/1471-2156-11-94 20950446 PMC2973851

[bib39] Kesari R. Lasky J. R. Villamor J. G. Marais D. L. D. Chen Y.-J. C., 2012 Intron-mediated alternative splicing of Arabidopsis P5CS1 and its association with natural variation in proline and climate adaptation. Proc. Natl. Acad. Sci. USA 109: 9197–9202. 10.1073/pnas.1203433109 22615385 PMC3384178

[bib40] Kimber, C. T., J. A. Dahlberg, and S. Kresovich, 2013 The Gene Pool of Sorghum bicolor and Its Improvement, pp. 23–41 in *Genomics of the Saccharinae*, edited by A. H. Paterson. Plant Genetics and Genomics: Crops and Models 11, Springer, New York.

[bib41] Kloosterman B. Abelenda J. A. Gomez Mdel M. Oortwijn M. de Boer J. M., 2013 Naturally occurring allele diversity allows potato cultivation in northern latitudes. Nature 495: 246–250. 10.1038/nature11912 23467094

[bib42] Korte A. Vilhjálmsson B. J. Segura V. Platt A. Long Q., 2012 A mixed-model approach for genome-wide association studies of correlated traits in structured populations. Nat. Genet. 44: 1066–1071. 10.1038/ng.2376 22902788 PMC3432668

[bib43] Kronholm I. Picó F. X. Alonso-Blanco C. Goudet J. de Meaux J., 2012 Genetic basis of adaptation in Arabidopsis thaliana: Local adaptation at the seed dormancy QTL DOG1. Evolution 66: 2287–2302. 10.1111/j.1558-5646.2012.01590.x 22759302

[bib44] Labeyrie V. Deu M. Barnaud A. Calatayud C. Buiron M., 2014 Influence of ethnolinguistic diversity on the sorghum genetic patterns in subsistence farming systems in eastern Kenya. PLoS One 9: e92178 10.1371/journal.pone.0092178 24637745 PMC3956919

[bib45] Lasky J. R. Des Marais D. L. McKay J. K. Richards J. H. Juenger T. E., 2012 Characterizing genomic variation of Arabidopsis thaliana: the roles of geography and climate. Mol. Ecol. 21: 5512–5529. 10.1111/j.1365-294X.2012.05709.x 22857709

[bib46] Lasky J. R. Upadhyaya H. D. Ramu P. Deshpande S. Hash C. T., 2015 Genome-environment associations in sorghum landraces predict adaptive traits. Sci. Adv. 1: e1400218 10.1126/sciadv.1400218 26601206 PMC4646766

[bib47] Leamy L. J. Lee C.-R. Song Q. Mujacic I. Luo Y., 2016 Environmental *vs.* geographical effects on genomic variation in wild soybean (Glycine soja) across its native range in northeast Asia. Ecol. Evol. 6: 6332–6344. 10.1002/ece3.2351 27648247 PMC5016653

[bib48] Leiser W. L. Rattunde H. F. Weltzien E. Cisse N. Abdou M., 2014 Two in one sweep: aluminum tolerance and grain yield in P-limited soils are associated to the same genomic region in West African Sorghum. BMC Plant Biol. 14: 206 10.1186/s12870-014-0206-6 25112843 PMC4256928

[bib49] Li H. Durbin R., 2009 Fast and accurate short read alignment with Burrows–Wheeler transform. Bioinformatics 25: 1754–1760. 10.1093/bioinformatics/btp324 19451168 PMC2705234

[bib50] Li L.-F. Li Y.-L. Jia Y. Caicedo A. L. Olsen K. M., 2017 Signatures of adaptation in the weedy rice genome. Nat. Genet. 49: 811–814. 10.1038/ng.3825 28369039

[bib51] Lipka A. E. Tian F. Wang Q. Peiffer J. Li M., 2012 GAPIT: genome association and prediction integrated tool. Bioinformatics 28: 2397–2399. 10.1093/bioinformatics/bts444 22796960

[bib52] Lobell D. B. Burke M. B. Tebaldi C. Mastrandrea M. D. Falcon W. P., 2008 Prioritizing climate change adaptation needs for food security in 2030. Science 319: 607–610. 10.1126/science.1152339 18239122

[bib53] Ma Y. Dai X. Xu Y. Luo W. Zheng X., 2015 COLD1 confers chilling tolerance in rice. Cell 160: 1209–1221. 10.1016/j.cell.2015.01.046 25728666

[bib54] Mace E. S. Li Y. Prentis P. J. Bian L. Campbell B. C., 2013 Whole-genome sequencing reveals untapped genetic potential in Africa’s indigenous cereal crop sorghum. Nat. Commun. 4: 2320 10.1038/ncomms3320 23982223 PMC3759062

[bib55] McCormick R. F. Truong S. K. Sreedasyam A. Jenkins J. Shu S., 2018 The Sorghum bicolor reference genome: improved assembly, gene annotations, a transcriptome atlas, and signatures of genome organization. Plant J. 93: 338–354. 10.1111/tpj.13781 29161754

[bib56] Meirmans P. G., 2015 Seven common mistakes in population genetics and how to avoid them. Mol. Ecol. 24: 3223–3231. 10.1111/mec.13243 25974103

[bib57] Menkir A. Goldsbrough P. Ejeta G., 1997 RAPD based assessment of genetic diversity in cultivated races of sorghum. Crop Sci. 37: 564–569. 10.2135/cropsci1997.0011183X003700020042x

[bib58] Meyer R. S. Choi J. Y. Sanches M. Plessis A. Flowers J. M., 2016 Domestication history and geographical adaptation inferred from a SNP map of African rice. Nat. Genet. 48: 1083–1088. 10.1038/ng.3633 27500524

[bib59] Meyer R. S. Purugganan M. D., 2013 Evolution of crop species: genetics of domestication and diversification. Nat. Rev. Genet. 14: 840–852. 10.1038/nrg3605 24240513

[bib60] Moles A. T. Warton D. I. Warman L. Swenson N. G. Laffan S. W., 2009 Global patterns in plant height. J. Ecol. 97: 923–932. 10.1111/j.1365-2745.2009.01526.x

[bib61] Molina J. Sikora M. Garud N. Flowers J. M. Rubinstein S., 2011 Molecular evidence for a single evolutionary origin of domesticated rice. Proc. Natl. Acad. Sci. USA 108: 8351–8356. 10.1073/pnas.1104686108 21536870 PMC3101000

[bib62] Morrell P. L. Buckler E. S. Ross-Ibarra J., 2012 Crop genomics: advances and applications. Nat. Rev. Genet. 13: 85–96. 10.1038/nrg3097 22207165

[bib63] Morris G. P. Ramu P. Deshpande S. P. Hash C. T. Shah T., 2013 Population genomic and genome-wide association studies of agroclimatic traits in sorghum. Proc. Natl. Acad. Sci. USA 110: 453–458. 10.1073/pnas.1215985110 23267105 PMC3545811

[bib64] Murphy R. L. Morishige D. T. Brady J. A. Rooney W. L. Yang S., 2014 Ghd7 (Ma6) represses sorghum flowering in long days: Ghd7 alleles enhance biomass accumulation and grain production. Plant Genome 7: 1–10. 10.3835/plantgenome2013.11.0040

[bib65] Myles S. Peiffer J. Brown P. J. Ersoz E. S. Zhang Z., 2009 Association mapping: critical considerations shift from genotyping to experimental design. Plant Cell 21: 2194–2202. 10.1105/tpc.109.068437 19654263 PMC2751942

[bib66] National Research Council, 1996 *Lost Crops of Africa: Volume I: Grains*. National Academy Press, Washington, D.C.

[bib67] Novembre J. Rienzo A. D., 2009 Spatial patterns of variation due to natural selection in humans. Nat. Rev. Genet. 10: 745–755. 10.1038/nrg2632 19823195 PMC3989104

[bib95] Nzeka U. Akhidenor J., 2018 Nigeria: Grain and Feed Annual. GAIN Reports, USDA-FAS. https://gain.fas.usda.gov/Recent GAIN Publications/Grain and Feed Annual_Lagos_Nigeria_4-12-2018.pdf

[bib68] Oksanen, J., F. G. Blanchet, M. Friendly, R. Kindt, P. Legendre *et al.*, 2017 *vegan: Community Ecology Package*.

[bib69] Olsen K. M., 2012 One gene’s shattering effects. Nat. Genet. 44: 616–617. 10.1038/ng.2289 22641204

[bib70] Paradis E. Claude J. Strimmer K., 2004 APE: analyses of phylogenetics and evolution in R language. Bioinformatics 20: 289–290. 10.1093/bioinformatics/btg412 14734327

[bib71] Pavlidis P. Jensen J. D. Stephan W. Stamatakis A., 2012 A critical assessment of storytelling: Gene ontology categories and the importance of validating genomic scans. Mol. Biol. Evol. 29: 3237–3248. 10.1093/molbev/mss136 22617950

[bib72] Rao K. E. P. Obilana A. T. Mengesha M. H., 1985 Collection of kaura, fara-fara and guineense sorghums in northern Nigeria. J. Agric. Tradit. Bot. Appl. 32: 73–81.

[bib73] Rellstab C. Gugerli F. Eckert A. J. Hancock A. M. Holderegger R., 2015 A practical guide to environmental association analysis in landscape genomics. Mol. Ecol. 24: 4348–4370. 10.1111/mec.13322 26184487

[bib74] Romero Navarro J. A. Willcox M. Burgueño J. Romay C. Swarts K., 2017 A study of allelic diversity underlying flowering-time adaptation in maize landraces. Nat. Genet. 49: 476–480. 10.1038/ng.3784 28166212

[bib75] Samis K. E. Murren C. J. Bossdorf O. Donohue K. Fenster C. B., 2012 Longitudinal trends in climate drive flowering time clines in North American Arabidopsis thaliana. Ecol. Evol. 2: 1162–1180. 10.1002/ece3.262 22833792 PMC3402192

[bib76] Savolainen O. Lascoux M. Merilä J., 2013 Ecological genomics of local adaptation. Nat. Rev. Genet. 14: 807–820. 10.1038/nrg3522 24136507

[bib77] Segura V. Vilhjálmsson B. J. Platt A. Korte A. Seren Ü., 2012 An efficient multi-locus mixed-model approach for genome-wide association studies in structured populations. Nat. Genet. 44: 825–830. 10.1038/ng.2314 22706313 PMC3386481

[bib78] Shanahan T. M. Overpeck J. T. Anchukaitis K. J. Beck J. W. Cole J. E., 2009 Atlantic forcing of persistent drought in West Africa. Science 324: 377–380. 10.1126/science.1166352 19372429

[bib79] Siol M. Wright S. I. Barrett S. C. H., 2010 The population genomics of plant adaptation. New Phytol. 188: 313–332. 10.1111/j.1469-8137.2010.03401.x 20696011

[bib80] Slatkin M., 1973 Gene flow and selection in a cline. Genetics 75: 733–756. 4778791 10.1093/genetics/75.4.733 PMC1213045

[bib81] Soler C. Saidou A.-A. Hamadou T. V. C. Pautasso M. Wencelius J., 2013 Correspondence between genetic structure and farmers’ taxonomy – a case study from dry-season sorghum landraces in northern Cameroon. Plant Genet. Resour. 11: 36–49. 10.1017/S1479262112000342

[bib82] Tuinstra M. R. Grote E. M. Goldsbrough P. B. Ejeta G., 1997 Genetic analysis of post-flowering drought tolerance and components of grain development in Sorghum bicolor (L.). Moench. Mol. Breed. 3: 439–448. 10.1023/A:1009673126345

[bib83] Vasconcelos A. C. F. Bonatti M. Schlindwein S. L. D’Agostini L. R. Homem L. R., 2013 Landraces as an adaptation strategy to climate change for smallholders in Santa Catarina, Southern Brazil. Land Use Policy 34: 250–254. 10.1016/j.landusepol.2013.03.017

[bib84] Vigouroux Y. Mariac C. Mita S. D. Pham J.-L. Gérard B., 2011 Selection for earlier flowering crop associated with climatic variations in the Sahel. PLoS One 6: e19563 10.1371/journal.pone.0019563 21573243 PMC3087796

[bib85] Westengen O. T. Okongo M. A. Onek L. Berg T. Upadhyaya H., 2014 Ethnolinguistic structuring of sorghum genetic diversity in Africa and the role of local seed systems. Proc. Natl. Acad. Sci. USA 111: 14100–14105. 10.1105/tpc.109.068437 25225391 PMC4191803

[bib86] Wu W. Zheng X.-M. Lu G. Zhong Z. Gao H., 2013 Association of functional nucleotide polymorphisms at DTH2 with the northward expansion of rice cultivation in Asia. Proc. Natl. Acad. Sci. USA 110: 2775–2780. 10.1073/pnas.1213962110 23388640 PMC3581972

[bib87] Yang S. Murphy R. L. Morishige D. T. Klein P. E. Rooney W. L., 2014 Sorghum phytochrome B inhibits flowering in long days by activating expression of SbPRR37 and SbGHD7, repressors of SbEHD1, SbCN8 and SbCN12. PLoS One 9: e105352 10.1371/journal.pone.0105352 25122453 PMC4133345

[bib88] Yoder J. B. Stanton-Geddes J. Zhou P. Briskine R. Young N. D., 2014 Genomic signature of adaptation to climate in Medicago truncatula. Genetics 196: 1263–1275. 10.1534/genetics.113.159319 24443444 PMC3982686

[bib89] Yohannes T. Abraha T. Kiambi D. Folkertsma R. Hash C. T., 2015 Marker-assisted introgression improves Striga resistance in an Eritrean farmer-preferred sorghum variety. Field Crops Res. 173: 22–29. 10.1016/j.fcr.2014.12.008

[bib90] Zeven A. C., 1998 Landraces: A review of definitions and classifications. Euphytica 104: 127–139. 10.1023/A:1018683119237

[bib91] Zhang D. Kong W. Robertson J. Goff V. H. Epps E., 2015 Genetic analysis of inflorescence and plant height components in sorghum (Panicoidae) and comparative genetics with rice (Oryzoidae). BMC Plant Biol. 15: 107 10.1186/s12870-015-0477-6 25896918 PMC4404672

[bib92] Zhen Y. Ungerer M. C., 2008 Clinal variation in freezing tolerance among natural accessions of Arabidopsis thaliana. New Phytol. 177: 419–427. 17995917 10.1111/j.1469-8137.2007.02262.x

[bib93] Zheng X. Levine D. Shen J. Gogarten S. M. Laurie C., 2012 A high-performance computing toolset for relatedness and principal component analysis of SNP data. Bioinformatics 28: 3326–3328. 10.1093/bioinformatics/bts606 23060615 PMC3519454

[bib94] Zhou X. Stephens M., 2014 Efficient multivariate linear mixed model algorithms for genome-wide association studies. Nat. Methods 11: 407–409. 10.1038/nmeth.2848 24531419 PMC4211878

